# Combined Band and Plate Fixation as a New Individual Option for Patients at Risk of Sternal Complications after Cardiac Surgery: A Single-Center Experience

**DOI:** 10.3390/biomedicines11071946

**Published:** 2023-07-10

**Authors:** Jules Miazza, Ion Vasiloi, Luca Koechlin, Brigitta Gahl, Oliver Reuthebuch, Friedrich S. Eckstein, David Santer

**Affiliations:** 1 Department of Cardiac Surgery, University Hospital Basel, 4031 Basel, Switzerland; jules.miazza@usb.ch (J.M.); ion.vasiloi@usb.ch (I.V.); luca.koechlin@usb.ch (L.K.); brigitta.gahl@usb.ch (B.G.); oliver.reuthebuch@usb.ch (O.R.); friedrich.eckstein@usb.ch (F.S.E.); 2 Faculty of Medicine, University of Basel, 4031 Basel, Switzerland

**Keywords:** cardiac surgery, sternotomy, sternal closure, SternaLock 360, osteosynthesis, sternal dehiscence, sternal mal-union

## Abstract

Due to the advent of interventional therapies for low- and intermediate-risk patients, case complexity has increased in cardiac surgery over the last decades. Despite the surgical progress achieved to keep up with the increase in the number of high-risk patients, the prevention of sternal complications remains a challenge requiring new, individualized sternal closure techniques. The aim of this study was to evaluate the safety and feasibility, as well as the in-hospital and long-term outcomes, of enhanced sternal closure with combined band and plate fixation using the new SternaLock^®^ 360 (SL360) system as an alternative to sternal wiring. From 2020 to 2022, 17 patients underwent enhanced sternal closure using the SL360 at our institution. We analyzed perioperative data, as well as clinical and radiologic follow-up data. The results were as follows: In total, 82% of the patients were treated with the SL360 based on perioperative risk factors, while in 18% of cases, the SL360 was used for secondary closure due to sternal instability. No perioperative complications were observed. We obtained the follow-up data of 82% of the patients (median follow-up time: 141 (47.8 to 511.5) days), showing no surgical revision, no sternal instability, no deep wound infections, and no sternal pain at the follow-up. In one case, a superficial wound infection was treated with antibiotics. In conclusion, enhanced sternal closure with the SL360 is easy to perform, effective, and safe. This system might be considered for both primary and secondary sternal closure in patients at risk of sternal complications.

## 1. Introduction

Sternotomy for surgical access was first described in 1897 [1]. Historically, steel wiring has been the method of choice to perform the sternal closure after cardiac surgery. Even though postoperative complications such as chronic pain, sternal mal-union, and postoperative deep wound infections are a source of physical and psychological stress for patients [2,3], and enhanced sternal closure techniques are recommended by the STS guidelines for patients undergoing bilateral internal thoracic artery harvesting [4], the traditional method of performing sternal closure has remained unchallenged over the years. In orthopedics, a discipline that is specialized in bone healing, osteosynthesis is routinely performed using rigid plates and screws instead of flexible wires [5]. In cardiac surgery, however, the most commonly used enhanced sternal closure option is the Robicsek technique [6], which is a combination of standard sternal wiring with additional reinforcement using lateral wires. Nevertheless, as shown in a prospective randomized multicenter trial by Schimmer et al., the Robicsek technique offers no advantages in terms of sternal dehiscence, superficial sternal wound infections, or deep sternal wound infections in high-risk patients when compared to standard sternal wiring [7].

In an attempt to reduce postoperative sternal complications, enhanced sternal closure using titanium plates has been proposed [8]. In both prospective [9] and retrospective [10] studies, enhanced sternal closure using sternal plates showed improved bone healing and reduced postoperative pain in patients deemed to be at high risk of postoperative sternal complications. Furthermore, a trend towards reduced postoperative wound infections has been observed [11].

**Figure 1:**
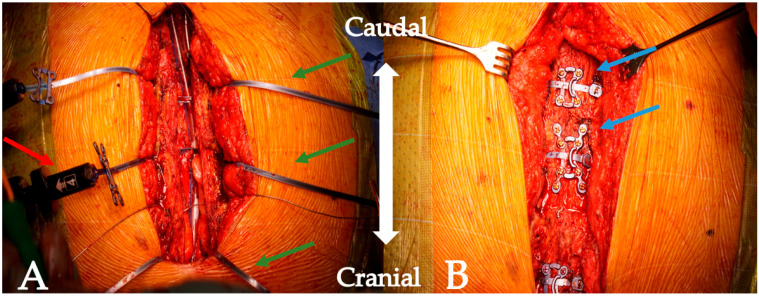
Intraoperative image showing the SL360 system (**A**) after passing the flexible bands through the sternum, as well as (**B**) after closure. Red arrow: incorporated tensioning system. Green arrows: the flexible sternal bands. Blue arrows: plate and screw after sternal closure.

In addition to single rigid plates, the SternaLock^®^ 360 system (SL360, Zimmer Biomet, Jacksonville, FL, USA) (Figure 1) provides a combination of rigid plates and a flexible sternal band to further reduce mechanical stress on frail sterna. The large width of the band compared to standard wiring was designed to improve the pressure repartition on the osteoporotic sternal bone. As Royse et al. [12] recently observed in the only published prospective study on the subject, the SL360 provides a reduced sternal edge motion at follow-up, in addition to a greater pain recovery at 6 and 12 weeks. Despite these promising results, the literature on enhanced sternal closure with the SL360 in patients after cardiac surgery remains scarce. Furthermore, it is noteworthy that the study by Royse et al. [12] relied on the data of unselected patients. While these results are precious, data on selected high-risk patients are lacking. The present study aimed to address the gap in the evidence by providing data on high-risk patients treated with the SL360. From May 2020 to July 2022, the incidence of sternal complications (sternal instability, sterile sternum dehiscence, or sternum infection) leading to reoperation during the same hospitalization time was 0.7% (8/1152) at our institution. The overall rate of reoperation due to sternal complication was 2.3% (26/1152). While the rate of sternal complications is low at our institution, the SL360 was introduced as a novel approach because patients who undergo cardiac surgery have become older and sicker over the last years [13,14]. The characteristics of the patients undergoing sternal reoperation are depicted in Supplemental Table S1.

In 2020, the SL360 was first introduced at the University Hospital of Basel, Basel, Switzerland. The aim of this study was to evaluate the outcomes of patients undergoing sternal closure with this new technique. To the best of our knowledge, this is the first retrospective single-center study with the SL360 based on intraoperative, surgeon-based decision making.

## 2. Materials and Methods

**Figure 2:**
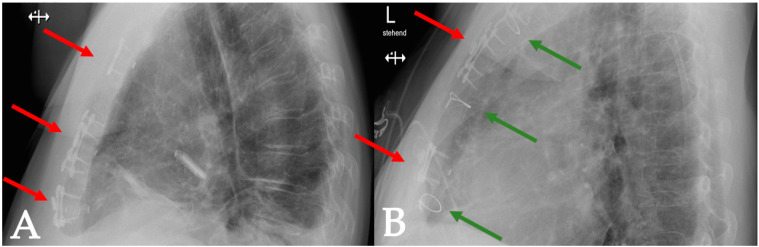
Comparison of enhanced sternal closure using the (**A**) SL360 alone (red arrows) or (**B**) SL360 (red arrows) in combination with three steel wires (green arrows).

A retrospective, single-center database analysis was performed at the Department of Cardiac Surgery, University Hospital of Basel, Switzerland. Seventeen patients underwent major cardiac surgery, followed by sternal closure using either the SL360 system alone or in combination with conventional sternal wires (Figure 2). Sternal closure using the SL360 was introduced at our institution in 2020 and has since been used in selected patients deemed at risk of sternal instability based on the surgeon’s intraoperative evaluation. The most common assessment for bone quality is the intraoperative sternum compressibility test [15], which is recommended by the manufacturer. The data presented in this study (patient characteristics, risk factors, intraoperative data, and outcomes) are routinely collected with prospectively maintained quality management software (Dendrite Clinical Systems, V1.7, Reading, UK) and regularly checked for completeness and consistency. The follow-up data were obtained from the patients’ medical records. In cases where follow-up was not available, the patients were excluded from the follow-up analysis. Imaging follow-up was not routinely performed but was included in patients undergoing computed tomography or magnetic resonance tomography for unrelated reasons. Superficial and deep wound infections were defined according to the Center for Disease Control and Prevention’s definition [16]. Sternal pain was defined as any pain in the operation site, with the exclusion of symptoms that could be associated with myocardial ischemia. Sternal mal-union was defined as a partial or total failure of fusion of the sternal halves, according to the available literature [17]. Sternal dehiscence was defined as a separation of the sternum halves, with symptoms of pain, instability, or consecutive reoperation [18]. The data are presented as the mean (standard deviation), median (quartiles), and number with percentage, according to the type and distribution of the data.

### 2.1. Surgical Technique

The SL360 system consists of three combined bands and plates (2 × 4 screw plates and 1 × 8 screw plate), each mounted on a tensioning system. The plates are analogous to the previous sternum closure system but are, in this case, completed with incorporated flexible bands. The first step of the implantation of the SL360 (Figure 1) is the measurement of the depth of the sternum using a designed tool. The measurements are performed at the level of the manubrium, as well as at the level of the 3rd and 5th intercostal spaces. Measurements are performed to find the optimal screw length to avoid loosening and complications due to perforation of the dorsal bone cortex by the screws [19]. To achieve a better sternum approximation, up to three single sternal wires can be added between the envisioned implantation sites of the SL360, thus allowing for a better positioning of the system. It is noteworthy that the use of additional single sternal wires primarily serves the purpose of achieving a better sternal approximation, while sternal stability is warranted by the SL360 system. This approach is recommended by the authors, especially for the first few cases with this new system. Alternatively, a Backhaus clamp can be used to achieve a better approximation. Next, the manubrium is perforated at its center using a bone punch, creating a path for the first flexible band. The remaining two flexible bands are passed through the 3rd and 5th intercostal spaces. Once the bands are placed (Figure 1A), the sternum is approximated stepwise using the incorporated tensioning system. At our institution, we also use the topical application of vancomycin paste before closure as a further prophylaxis for sternal infection [20]. Once the approximation of the sternum is achieved, the tensioning system is used to lock and cut the flexible bands. Finally, the rigid plates can be bent and adapted to mimic the anatomy of the sternum and are then fixed with screws (Figure 1B). In case of urgent re-thoracotomy, the system, including the plates, can be cut open using a standard wire cutter, preferably with an angulated cutting tip.

### 2.2. Patient Selection

The decision to perform enhanced sternal closure was based on the evaluation of preoperative and intraoperative risk factors, as well as on intraoperative bone assessments. In an attempt to facilitate decision making regarding multimorbid patients undergoing cardiac surgery, Nooh et al. [21] developed a sternal mal-union prediction scale (MUST-Score). The MUST-Score is a preoperative assessment tool based on predictors derived from data from over seven thousand patients who have undergone sternotomy. The score aims to predict the risk of postoperative sternal mal-union leading to reoperation. The MUST-Score ranges from 0 to >18 points, corresponding to a low (0 to 4 points), intermediate (4 to 8 points), high (8 to 11 points), very high (11 to 14 points), and extremely high (14 to >18 points) risk level. According to the risk stratification provided by the MUST-Score, the authors recommend a stepwise escalation in sternal closure techniques, ranging from single wires for low-risk patients to enhanced sternal closure techniques for patients at a higher risk [21]. In our cohort, the MUST-Score was calculated for all patients. Moreover, the decision to perform enhanced sternal closure was based on an intraoperative bone assessment. At our institution, sternal bone assessment is based on the sternum compressibility test [15], which is recommended by the manufacturer. The anterior and posterior sternal bones are pushed towards each other with the thumb and index finger. Healthy sternal bone should show little to no compliance. In cases where the sternal bone can be easily compressed, the test is considered pathological and enhanced sternal closure is suggested. Further indications for using the SL360 include bilateral internal mammary artery harvesting in the presence of diabetes mellitus increasing the risk of postoperative wound complications, the surgeon’s preferences, and sternal instability following cardiac surgery procedures over six months prior to using the SL360. 

## 3. Results

### 3.1. Preoperative Patient Characteristics

Between May 2020 and July 2022, seventeen patients underwent combined band and plate fixation at our institution. The mean (SD) age was 71 (6.2), and 65% were female. The median body mass index was 27 (24 to 31) kg/m^2^. All of the patients were over 60 years of age, and the preoperative MUST-Score [21] was 4.0 (3.0 to 6.0) points, representing an intermediate risk (1.4%) of postoperative sternal mal-union. The demographic data are presented in Table 1.

### 3.2. Operative Data

In 82% of the patients (*n* = 14), the SL360 was used at the time of the index heart operation, while in 18% (*n* = 3), the SL360 was used due to sternal instability at a later point in time. In patients receiving treatment with the SL360 at the time of the index surgery, 53% (*n* = 9) underwent isolated coronary artery bypass grafting, 18% (*n* = 3) underwent combined procedures, 12% (*n* = 2) underwent an operation on the aorta, 6% (*n* = 1) underwent a valve procedure only, 6% (*n* = 1) underwent left ventricular assist device (LVAD) implantation, and 6% (*n* = 1) were operated on due to pericardial disease. Intraoperative bone assessments using the sternum compressibility test were pathological in 47% of cases (*n* = 8). The operative data are depicted in Table 2. An overview of the factors leading to the use of the SL360 is provided in Table 3.

### 3.3. Postoperative Outcomes

No operative mortality was observed among the postoperative outcomes. The median (IQR) length of intensive care unit stay was 2.0 (1.0 to 2.0) days. There were no cases of reoperation for bleeding, and no cases of sternal infection. The median (IQR) length of hospital stay was 9.0 (8.0 to 11.0) days. The postoperative outcomes are depicted in Table 4.

### 3.4. Follow-Up Outcomes

**Figure 3:**
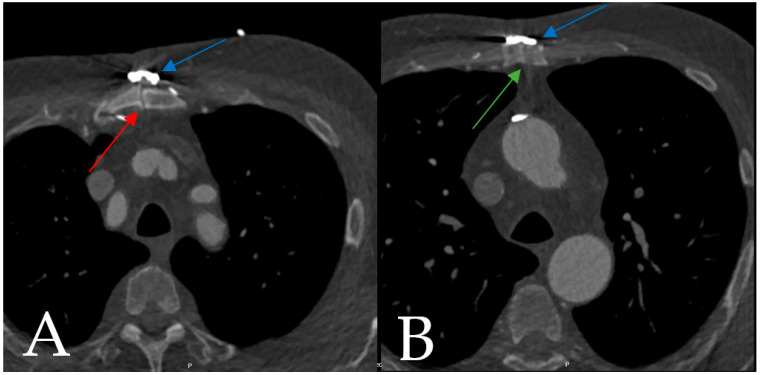
Postoperative computed tomography of a 76-year-old male 3 months after aortic valve replacement and replacement of the ascending aorta, as well as enhanced sternal closure using the SL360. (**A**) Red arrow: 3 mm mal-union zone in the region of the manubrium sterni. (**B**) Green arrow: consolidated bone in the region of the 3rd intercostal space. (**A**,**B**) Blue arrow: SL360 enhanced sternal closure system.

The clinical follow-up was completed in 82% (*n* = 14) of the patients, with a median (IQR) follow-up time of 141 (47.8, 511.5) days. One patient was excluded from the follow-up after SL360 explantation and conventional sternal closure in the context of a revision for bypass occlusion. At follow-up, there were no deep wound infections, no cases of sternal instability, and no revisions linked to the combined band and plate fixation. One patient (6%) developed a postoperative superficial wound infection, which was treated with co-amoxicillin (see Section 4.1.1). Six patients (35%) underwent imaging at the time of the follow-up. In one case, a 3 mm partial mal-union of the cranial sternum (Figure 3) was reported at the 3-month follow-up. In all of the other cases, no sternal mal-union was reported. The results are detailed in Table 5.

## 4. Discussion

In this retrospective, descriptive, single-center study, we provide some of the first real-world data on the use of the new band and plate fixation system SL360 as an alternative to standard sternal wiring after cardiac surgery. In our cohort, this technique was shown to be safe and feasible in all of the patients. We report no cases of postoperative sternum instability, deep wound infection, or reoperation linked to sternal complications during the hospital stay or at the follow-up. At follow-up, no patient reported sternal pain. Furthermore, the imaging data revealed one case of cranial sternal mal-union (3 mm) without any sign of instability. In all of the other patients, complete sternal ossification was observed.

According to the literature, the risk factors for postoperative sternal complications are either patient-related (smoking, lung disease, obesity, diabetes mellitus, renal failure, and osteoporosis) or procedure-related (off-midline sternotomy, bilateral mammary artery harvesting, and prolonged cardiopulmonary bypass time) [3,22,23]. In our cohort, the median preoperative MUST-Score was four points (IQR 3.0 to 6.0), representing an intermediate risk of sternal mal-union (1.4%). Despite this acceptable risk, enhanced sternal closure was performed on all of the patients driven by intraoperative sternum inspection. These results emphasize the importance of an intraoperative bone quality assessment. Furthermore, the MUST-Score has not been externally validated yet. Although only 12% of the patients had been diagnosed with osteoporosis or osteopenia in our cohort, the surgeons reported a pathological sternal compressibility test in 47% of the cases, leading to the decision to perform enhanced sternal closure.

The aim of enhanced sternal closure is to reduce postoperative sternal complications, mainly comprising sternal pain, sternal infections, and sternal mal-union and instability. Chronic post-sternotomy pain is a complication that occurs in 28% of patients, with up to one third of patients reporting sleep disturbances due to pain [3,24]. In order to reduce the incidence of this postoperative burden, surgeons continuously seek perioperative causes of postoperative pain [25], including sternal stability. The only available prospective data on enhanced sternal closure with the SL360 show markedly higher pain recovery with the SL360 when compared to standard wiring. This correlates with our cohort, in which no patient reported pain at the follow-up. Therefore, enhanced sternal closure might help to pave the way toward increased freedom from sternal pain after cardiac surgery.

Postoperative deep wound infection after cardiac surgery is a serious and potentially life-threatening complication, with an incidence ranging from 1% to over 10%, according to various studies [26,27,28,29]. The therapeutic approaches to such complications range from antibiotic therapy to pectoralis flap procedures, depending on its severity [26]. In our cohort, we report no cases of postoperative deep wound infection, and one case of postoperative superficial wound infection requiring antibiotic therapy. In order to evaluate the potential of the SL360 for the prophylaxis of postoperative wound infections, further studies comparing this technique to sternal wiring are urgently needed.

Postoperative sternum instability requiring reoperation after cardiac surgery occurs in 0.8% to 10% of patients [30,31,32,33]. In our cohort, no sternum instability was reported at the follow-up. These findings are congruent with the work of Royse et al. [12], showing reduced sternal edge motion in patients treated with the SL360 compared to standard wiring. In one patient, a 3 mm mal-union on the cranial part of the sternum was diagnosed. Despite this finding, the patient reported no pain, and the clinical examination showed no sternal instability during the postoperative course or at the follow-up. Since computed tomography was not performed during the index hospitalization, the mal-union was only diagnosed at follow-up, and was interpreted as iatrogenic due to an insufficient approximation during implantation. Considering that the patient was asymptomatic, no revision was needed.

In three patients, the SL360 was used due to sternal instability at least 6 months after the index surgery. In these patients, the SL360 provided enhanced clinical sternal stability and freedom from pain at the follow-up.

The incidence of re-exploration after cardiac surgery (e.g., due to bleeding complications) ranges from 2.2 to 4.2% [34]; therefore, sternal closure systems need to be reopened easily. In the case of the SL360, the titanium plate and the flexible bands are designed to allow cutting using a conventional wire cutter with an angulated tip. In our cohort, re-exploration was performed in one patient due to bypass occlusion. The SL360 was deemed easy to reopen without a prolonged surgery time, which has been reported by Royse et al. as well [12]. It is noteworthy that data on long-term reoperation after SL360 use, as well as data on the use of SL360 in patients with previous sternotomies, are lacking and that studies are urgently needed considering the potential utility of enhanced sternal closure techniques for this delicate patient cohort [35].

The decision to perform enhanced sternal closure should be based on a broad assessment of patient- and surgery-related factors in order to provide individualized care for cardiac surgery patients. In their paper on the development of a new sternal dehiscence prediction scale, Nooh et al. [21] proposed the escalation of sternal closure techniques according to their risk score, ranging from standard sternal wiring for patients with a low risk of sternal dehiscence up to enhanced sternal closure using the SL360 for patients with a high risk of postoperative sternal dehiscence. Consecutively, it is recommended to use standard sternal wiring using at least eight wires for patients with a low risk of sternal dehiscence, as it has been shown to be safe and efficient in the literature [36]. For patients with an intermediate-to-high risk of sternal dehiscence, enhanced sternal closure should be pursued. Enhanced sternal closure can be performed either by using a combination of a single sternal plate with wires or, as in our cohort, a combined band and plate fixation system. While single plating has been shown to reduce postoperative sternal complications [11], data on enhanced sternal closure using combined band and plate fixation are scarce. However, as Royse et al. showed in a prospective study in 2021, the use of the SL360 contributes to reduced sternal edge motion and a greater recovery from pain. These results are congruent with those reported in our study.

At our institution, we use a clinical decision-making approach based on preoperative risk factors, intraoperative bone assessments (sternum compressibility test), and surgical characteristics (BIMA harvesting). In addition, a tailored approach for sternal closure allows us to address our patients’ individual demands for postoperative rehabilitation (see Section 4.1.2). The presented results are encouraging and underline the therapeutic potential of the SL360 for enhanced sternal closure. In the past, enhanced sternal closure has been performed using the SternaLock^®^ Blu system (SternaLock Blu, Zimmer Biomet, Jacksonville, FL, USA), which is composed of rigid plates without flexible bands and has shown reduced sternal complications [11]. However, as the literature shows [13,14], the risk profile of patients undergoing cardiac surgery has been continuously increasing over the last decades. In particular, an increase in the incidence of diabetes and obesity has been observed, both of which are known risk factors for sternal complications [13]. These demographic changes create the need for further innovation in the field of sternal osteosynthesis. Considering the promising results of this single-center study, the SL360 might complete the toolbox of cardiac surgeons for enhanced and individualized sternal closure.

### 4.1. Individualized Therapy

As well as the use of the SL360 in high-risk patients, we also present two patients who demanded maximum postoperative sternal stability and, therefore, individualized concepts for sternal closure due to their comorbidities.

#### 4.1.1. Case 1

**Figure 4:**
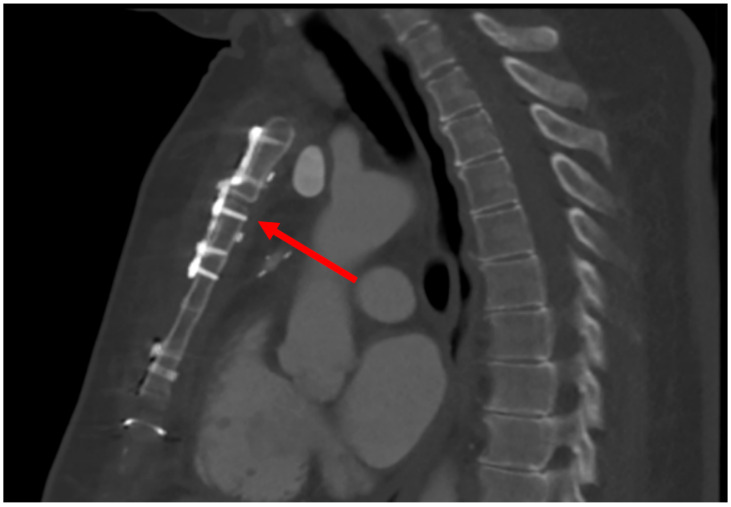
Computed tomography on postoperative day 21. The red arrow points to the tip of the screw reaching the posterior sternum lamella, demonstrating the importance of the careful measurement of screw lengths, as described in Section 2.1.

We implanted the SL360 in a 64-year-old female patient with a BMI of 37 kg/m^2^ and a history of diabetes mellitus with HbA1c of 9.5%. The patient underwent urgent coronary artery bypass grafting due to a non-ST-elevation myocardial infarction. The preoperative MUST-Score for this patient was 10 points, representing a risk of 12.2% for postoperative sternal mal-union. In order to reduce the risk of postoperative sternum complications, we decided to use the SL360 for primary closure. The patient presented at our emergency department on postoperative day 21 with a superficial wound infection, which was successfully treated with antibiotics. The imaging of the sternum with computed tomography (Figure 4) showed no dehiscence and beginning ossification.

#### 4.1.2. Case 2

**Figure 5:**
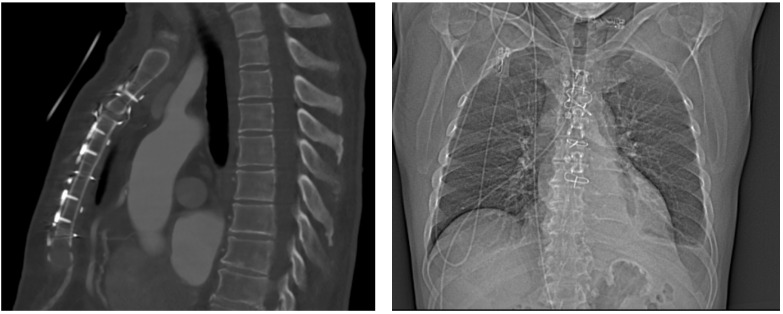
Enhanced sternal closure in a patient with a history of cerebral palsy since childhood.

A 65-year-old male patient underwent coronary artery bypass grafting due to coronary multi-vessel disease. The patient had a history of cerebral palsy since childhood and was relying mostly on crutches and a wheelchair for his mobility. He lived alone and was independent in all daily activities. Since the patient was wheelchair-bound, the operating team decided to perform primary sternal closure with the SL360 (Figure 5) in order to increase sternal stability and allow full rehabilitation. This individualized therapy helped to preserve the patient’s autonomy while being able to provide full surgical revascularization. The patient was discharged into the rehabilitation facility on postoperative day seven while already using a wheelchair without pain or signs of sternal instability.

### 4.2. Limitations

This study has several limitations. First, it is a retrospective, single-center analysis with a small number of participants. The relatively high costs [11] and selected patient population benefiting from the SL360 are indeed prohibitive for the systematic implantation of the system. Second, this study only provides a descriptive analysis of patients treated with the SL360. Furthermore, imaging follow-up was not routinely performed, and an objective measure of sternal stability, such as ultrasound-measured sternal edge motion, was not available. Further studies providing imaging follow-up and standardized quantification of sternal instability are needed in order to compare the efficacy of the SL360 in reducing postoperative sternal mal-union when compared to rigid plate fixation and conventional sternal wiring.

Considering that postoperative sternal instability and postoperative deep wound infections are relatively rare conditions occurring in 0.8% to 10% [30,31,32,33] and 1% to 10% [26,27,28,29] of patients, respectively, the sample size of this cohort was not sufficient enough to draw definitive conclusions on enhanced sternal closure with the SL360 after cardiac surgery. Third, even though the follow-up rate was 82%, sternal imaging is not routinely performed at our institution and was only available in 35% of the patients. Imaging studies are needed in order to analyze the ossification of the sternum after use of the SL360.

## 5. Conclusions

In this retrospective, single-center analysis, we report the safety and feasibility of the use of the SL360 as a new option for enhanced sternal closure. To our knowledge, this is the first retrospective report of the successful implementation of band and plate fixation for sternal closure. This study provides real-world data with surgeon-based, intraoperative decision making. The follow-up showed no postoperative revisions, no deep wound infections, no sternal instability, and no sternal pain. We also presented a case of individualized therapy with the SL360 that allowed for immediate rehabilitation in a patient depending on crutches for mobility. The recent literature has already shown that the SL360 is a safe alternative to sternal (8) wiring, and our study confirms these results. Due to the growing demand for individualized therapy in multimorbid patients, the SL360 seems to be a reasonable addition to standard wiring, as well as plating, for sternal closure in complex and high-risk patients, next to conventional wiring and rigid plate fixation. Prospective studies are needed in order to compare the SL360 with sternal plates and to identify high-risk patients who may benefit the most from this technique.

## Figures and Tables

**Table 1:** Preoperative patient characteristics.

Patient Characteristics	Total (N = 17)
Age, years	71 (6.2)
Female	11 (65%)
Diabetes	5 (29%)
No insulin	2 (12%)
Insulin	3 (18%)
HbA1c, %	5.7 (5.4 to 7.2)
BMI kg/m^2^	27 (24 to 31)
BMI > 35 kg/m^2^	3 (18%)
BMI 25–35 kg/m^2^	9 (53%)
3-Vessel CAD	8 (47%)
Left main CAD	2 (12%)
Peripheral artery disease	1 (6%)
Preoperative stroke	3 (18%)
Renal disease	1 (6%)
Dialysis	0 (0%)
COPD	2 (12%)
Prior myocardial infarction	12 (71%)
Hypertension	13 (76%)
Hypercholesteremia	7 (41%)
Diagnosed with osteoporosis or osteopenia	2 (12%)
Smoker	4 (24%)
Current	3 (18%)
Former	1 (6%)
Previous cardiac operations > 6 months	2 (12%)
MUST-Score	4 (3.0 to 6.0)
Dyspnea grade NYHA	
n/a	1 (6%)
I	14 (82%)
II	2 (12%)
Dyspnea NYHA III or IV	0 (0%)
Preoperative AF	2 (12%)
Left ventricular ejection fraction, %	52 (12)

Data are presented as the mean and standard deviation, median and interquartile range, or number and %. AF—atrial fibrillation; BMI—Body mass index; CAD—coronary artery disease; COPD—chronic obstructive pulmonary disease; MUST-Score—sternal mal-union prediction scale; NYHA—New York Heart Association.

**Table 2:** Operative data.

Operative Data	Total (N = 17)
CABG and Valve(s)	3 (18%)
CABG only	9 (53%)
Valve(s) only	1 (6%)
Aorta	2 (12%)
LVAD	1 (6%)
Pericardial disease	1 (6%)
Emergency	1 (6%)
Re-exploration	2 (12%)
EuroSCORE II	3.3 (1.4 to 6.6)
Duration of operation	223 (177 to 278)
Duration of surgery > 300 min	1 (6%)
Perfusion time, min	108 (82 to 133)
Aortic clamping time, min	75 (45 to 87)
Indications for SL360	
MUST-Score > 8	4 (24%)
Intraoperative osteoporosis/	
pathological sternal compressibility test	8 (47%)
BIMA and diabetes [4]	1 (6%)
Surgeon’s preference	2 (12%)
Sternal instability	3 (18%)
Secondary sternal closure	3 (18%)
SL360 only	2 (12%)
SL360 and sternal wires	15 (88%)
SL360 at index operation	14 (82%)
SL360 as secondary procedure	3 (18%)

Data are presented as the median and interquartile range or number and %. BIMA—bilateral internal mammary artery harvesting; CABG—coronary artery bypass grafting; LVAD—left ventricular assist device; SL360—SternaLock 360.

**Table 3:** Patient characteristics and factors leading to SL360 implantation.

Patient	History of Diabetes	Preoperative HbA1 (%)	BIMA	History of Osteoporosis or Osteopenia	BMI (kg/m^2^)	Secondary Sternal Closure	Pathological Sternal Compressibility Test	Surgeon’s Preference for SL360	Age > 60 Years
1	NIDDM	7.3	X		32				X
2		5.4			28			X	X
3		6.7			21		X		X
4	IDDM	6			26				X
5		5.7	X	X	28		X		X
6		n.a.		X	40		X		X
7		n.a.			n.a.		X	X	X
8	IDDM	7.6			39				X
9	NIDDM	7.8			29	X			X
10		5.7			31	X			X
11		n.a.			22				X
12		5.1	X		26	X			X
13		5.2			27		X		X
14	IDDM	9.5			37				X
15		5.5			24		X		X
16		5.3			20		X		X
17		5.6			21		X		X

NIDDM—non-insulin-dependent diabetes mellitus; IDDM—insulin-dependent diabetes mellitus; n.a.—not available.

**Table 4:** Postoperative outcomes.

Postoperative Outcomes	Total (N = 17)
Operative mortality	0 (0%)
ICU stay, d	2.0 (1.0 to 2.0)
Reoperation for bleeding	0 (0%)
Postoperative myocardial infarction	1 (6%)
Postoperative stroke	2 (12%)
Time to postoperative mobilization into standing position, d	2 (2 to 3)
Atrial fibrillation at discharge	3 (18%)
Permanent pacemaker	0 (0%)
Sternal infection	0 (0%)
Postoperative renal failure	2 (12%)
Renal substitution therapy	0 (0%)
Pulmonary infection	0 (0%)
MACCE	3 (18%)
Sepsis	1 (6%)
Length of in-hospital stay, d	9.0 (8.0 to 11)

Data are presented as the median and interquartile range or number and %. ICU—intensive care unit; MACCE—major adverse cerebral and cardiovascular events.

**Table 5:** Follow-up.

Outcomes at Follow-Up	Total (N = 17)
Available follow-up	14 (82%)
Follow-up time, days	141 (47.8 to 511.5)
Deep wound infection	0 (0%)
Superficial wound infection	1 (6%)
Sternal instability	0 (0%)
Sternal pain	0 (0%)
Revision linked to SL360	0 (0%)
CT scan	6 (35%)
Sternal dehiscence	0 (0%)
Sternal mal-union	1 (6%)

Data are presented as the median and interquartile range or number and %. CT—computed tomography.

## Data Availability

The datasets used and/or analyzed during the current study are available from the corresponding author on reasonable request.

## Supplementary Materials

The following supporting information can be downloaded at: https://www.mdpi.com/article/10.3390/biomedicines11071946/s1, Table S1: Baseline characteristics of patients undergoing sternal reoperation.
