# Supplementary material for: Combined Band and Plate Fixation as a New Individual Option for Patients at Risk of Sternal Complications after Cardiac Surgery: A Single-Center Experience
Source: Biomedicines. 2023 Jul 10;11(7):1946. doi: 10.3390/biomedicines11071946 (PMC10377508; doi:10.3390/biomedicines11071946)
Supplement: Supplementary file 1 [file biomedicines-11-01946-s001.zip › biomedicines-2402133-SI.pdf]

Supplemental Table S1: Baseline Characteristics of patients undergoing sternal reoperation

Patient characteristics	Total (N = 26)
Age, years	64 (59 to 70)
Female	12 (46%)
Diabetes	
No insulin	3 (11.5%)
Insulin	4 (15%)
BMI	29 (24 to 34)
3-vessel CAD	9 (35%)
Left main CAD	4 (15%)
Peripheral artery disease	0 (0%)
Preoperative stroke	0 (0%)
Renal disease	7 (27%)
Dialysis	0 (0%)
COPD	3 (12%)
Prior myocardial infarction	9 (35%)
Hypertension	22 (85%)
Hypercholesterolemia	9 (35%)
Current smoker	11 (42%)
Dyspnea NYHA III or IV	2 (7.7%)
Atrial fibrillation	2 (7.7%
Left ventricular ejection fraction, %	55 (50 to 61)
EuroSCORE II	4.1 (1.9 to 6.9)

Patient characteristics of patients who underwent reoperation for sternal indication during enrolment, N=26. Time since index surgery was 151 days on average and ranged from 5 days to 19 months.
Data are presented as the mean and standard deviation, median and interquartile range, or number and %.
AF—atrial fibrillation; BMI – body mass index; CAD—coronary artery disease; COPD—chronic obstructive pulmonary disease; NYHA—New York Heart Association.
